# Examining the cost-effectiveness of personal protective equipment for formal healthcare workers in Kenya during the COVID-19 pandemic

**DOI:** 10.1186/s12913-021-07015-w

**Published:** 2021-09-20

**Authors:** Jacob Kazungu, Kenneth Munge, Kalin Werner, Nicholas Risko, Andres I. Vecino-Ortiz, Vincent Were

**Affiliations:** 1 Health Economics Research Unit, Kenya Medical Research Institute -Wellcome Trust, P.O Box 43640-0010, Nairobi, Kenya; 2 The World Bank, Kenya Country Office, Nairobi, Kenya; 3 The University of Cape Town, Cape Town, South Africa; 4 Johns Hopkins Bloomberg School of Public Health International Health Department, Johns Hopkins University School of Medicine, Baltimore, MD USA

**Keywords:** Cost-effectiveness, Return on investment, PPE, Healthcare workers, Front-line workers, COVID-19

## Abstract

**Background:**

Healthcare workers are at a higher risk of COVID-19 infection during care encounters compared to the general population. Personal Protective Equipment (PPE) have been shown to protect COVID-19 among healthcare workers, however, Kenya has faced PPE shortages that can adequately protect all healthcare workers. We, therefore, examined the health and economic consequences of investing in PPE for healthcare workers in Kenya.

**Methods:**

We conducted a cost-effectiveness and return on investment (ROI) analysis using a decision-analytic model following the Consolidated Health Economic Evaluation Reporting Standards (CHEERS) guidelines. We examined two outcomes: 1) the incremental cost per healthcare worker death averted, and 2) the incremental cost per healthcare worker COVID-19 case averted. We performed a multivariate sensitivity analysis using 10,000 Monte Carlo simulations.

**Results:**

Kenya would need to invest $3.12 million (95% CI: 2.65–3.59) to adequately protect healthcare workers against COVID-19. This investment would avert 416 (IQR: 330–517) and 30,041 (IQR: 7243 – 102,480) healthcare worker deaths and COVID-19 cases respectively. Additionally, such an investment would result in a healthcare system ROI of $170.64 million (IQR: 138–209) – equivalent to an 11.04 times return.

**Conclusion:**

Despite other nationwide COVID-19 prevention measures such as social distancing, over 70% of healthcare workers will still be infected if the availability of PPE remains scarce. As part of the COVID-19 response strategy, the government should consider adequate investment in PPE for all healthcare workers in the country as it provides a large return on investment and it is value for money.

## Introduction

On March 11, 2020, the World Health Organization (WHO) declared coronavirus 2019 (COVID-19) a global pandemic. The WHO estimates that 10% of the global COVID-19 clinically diagnosed cases are among health workers with over 10,000 of the infected health workers coming from 40 African countries [1]. Health care workers have been reported to have 11.7 times the risk of testing positive for COVID-19 compared to the general community [2]. This increased risk of infection has been primarily attributed to a lack of adequate personal protective equipment (PPE) [3, 4].

Although existing evidence indicates that the type of PPE may determine the level of protection against COVID-19 infection among healthcare workers [5], there is a consensus for the consistent use of PPEs (a surgical mask, gloves, eye protection and a gown) when providing care for COVID-19 patients [2, 6]. Providing PPE to healthcare workers is, therefore, a critical component of the response to the COVID-19 pandemic [7, 8].

Countries are experiencing PPE shortages for frontline health care workers. PPE availability is affected by increased demand, global supply chain disruptions resulting from interventions to control the pandemic [9], challenges with ensuring rational use, ensuring supplies are preserved for areas with the greatest need, and lack of accountability in delivering PPE supplies to the frontline. This is despite the growth in local manufacturing of PPE in many countries and gradual improvements in international supply chains [10].

Ensuring healthcare workers are protected from COVID-19 infection is paramount especially in those countries with low healthcare worker numbers in absolute and relative terms [11]. These same countries will experience greater strain on their ability to manage cases of COVID-19.

Kenya is a lower-middle-income country with 13.8 health care workers per 10,000 population in 2016. There is a steady pipeline of human resources for health development mainly from non-university tertiary level institutions [12, 13]. The workers are employed in both public and private sectors with dual practice common [14]. Public sector employment is mainly through county governments who are the main providers of public health services [15]. Private sector employment is through not-for-profit and for-profit organizations and sole proprietorships [16]. Healthcare workers are inequitably distributed with urban areas attracting and retaining more. There are challenges with the effective management of public sector workers contributing to repeated episodes of industrial action in recent years [17].

Kenya reported 3068 infections and 32 mortalities among healthcare workers as of 11th January 2021 [18]. This represents 3.1% of total infections as of this date. Anecdotal evidence identifies the availability of PPE as a key contributor to these infections. Concerns about the availability of PPE in Kenya have led to industrial unrest among healthcare workers [19]. The impacts of continued infection of healthcare workers are likely to be severe but are yet unknown. This paper seeks to quantify the costs and cost-effectiveness of availing adequate PPE to healthcare workers in Kenya and the resulting return on investment.

## Methods

**Fig. 1:**
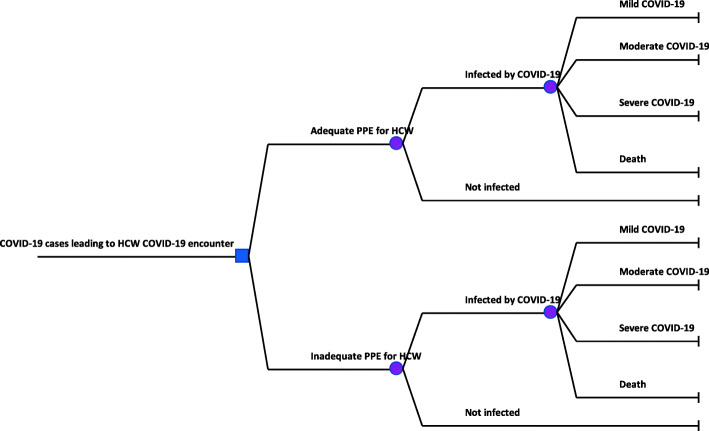
Decision tree model

For this analysis, we adopted an approach described elsewhere [20] to generate country-specific estimates for Kenya. In summary, we developed a decision-analytic model (Fig. 1) to compare the costs and effects of two PPE use scenarios in Kenya following the Consolidated health economic evaluation reporting standards (CHEERS) guidelines [21].

We compared a scenario where adequate/full PPE utilisation reduces healthcare worker infection and mortality to a scenario where healthcare workers had an inadequate supply of PPE thus higher rates of infection and mortality. PPE was considered adequate if healthcare workers had access to gloves, gown, surgical masks, and face shields, otherwise, a lack of either of these was considered as inadequate supply of PPE. Two outcomes were examined: 1) the incremental cost per healthcare worker death averted and 2) the incremental cost per healthcare worker case averted.

We then performed a Return on Investment (ROI) analysis that compared the health system economic benefits from having all healthcare workers protected against COVID-19 infection to the investment required to afford the PPE.

Several data sources and approaches were adopted for this analysis. Our definition of healthcare workers includes those recorded as nurses, clinical officers, doctors and lab technologists in the Statistical Abstract [13].

First, we utilised the WHO COVID-19 Essential Supplies Forecasting Tool (ESFT) to estimate the costs and required resources [22]. The projections represent a 30-week period starting March 2021 following the WHO guideline on PPE requirements [23].

Second, healthcare worker labour costs were abstracted from a salary survey in four counties in Kenya (data not yet published) whereas costs for utilization of COVID-19 services were adopted from a cross-sectional study in Kenya [24]. Costs are presented in 2020 US dollars from a health system perspective. We used an exchange rate of 1 USD = KES 108.43 to convert the costs into 2020 US dollars. In the model these costs also accounted for the healthcare worker lost productivity resulting from an early death or hospitalization. Our study also included training costs (unlike in the global study – Risko et al., [20]) for the different cadres of healthcare workers in Kenya. Training costs included the tuition fees, living expense and costs for obtaining required licences to practice for the different cadres of healthcare workers included in this study [25, 26]. Costs/benefits were not discounted as the investment would be required to be done over a short period of less than 1 year. Healthcare worker premature death was estimated to result in the loss of 16 years of working life (calculated as the difference between retirement age – 60 years – and the average age of a healthcare worker – 44 years [27]. In Kenya, healthcare workers are comprised of doctors, nurses, clinical officers, technicians and ancillary staff. Table 1 summarises the main parameter values, their ranges, distribution and sources.

**Table 1:** Main model parameters

Parameter	Value	Distribution	Source
***Epidemiologic Variables***			
Kenyan deaths due to COVID19	8914 (101–36,864)	lognormal	[28]
Kenyan COVID19 cases (thousands)	645.97 (7.35–2671.33)	lognormal	[28]
HCW infections as % of total infections (*full PPE case*)	0.034 (0.029–0.039)	beta	Estimate, [12]
HCW infections as % of total infection (*limited PPE case*)	0.05 (0.04–0.06)	beta	[29]
Case acuity mix % (*mild/moderate/critical*)	80.0/13.8/6.20	beta	[30]
Case fatality (%)	1.38 (1.23–1.53)	beta	[31]
***Utilization Inputs***	**Value (range for sensitivity analysis)**		
Mean hospital days for severe infection	11 (6–21)	lognormal	[32]
Days of work missed for infection (*mild/moderate/severe*)	13/28/40	lognormal	[32]
***Cost Inputs (2020 USD)***			
Cost of training a Medical Officer	31,541 (26,810-36,272)	gamma	, ^b^
Cost of training a clinical officer	6660 (5661-7659)	gamma	[33, 34], ^b^
Cost of training a nurse	6660 (5661-7659)	gamma	[33, 34], ^b^
Cost of training a laboratory technologist	4858 (4130-5587)	gamma	[33, 34], ^b^
Cost per Doctor-day of work	102 (87–118)	gamma	^a^
Cost per clinical officer-day of work	51 (43–58)	gamma	^a^
Cost per nurse-day of work	44 (38–51)	gamma	^a^
Cost per lab tech-day of work	34 (29–39)	gamma	^a^
Cost of supplies (millions)	3.12 (2.65–3.59)	gamma	[35]
Hospital bed per day	30.26 (8.17–52.35)	gamma	[24, 35]
GDP per capita	1817 (1544-2088)	gamma	
Number of HCW	161,160	lognormal	[12]

^a^ Estimates from a salary survey in four counties in Kenya (data not yet published); ^b^ Estimate for living expense and licence

### Sensitivity analysis

Third, we performed probabilistic sensitivity (PSA) analyses to examine how a simultaneous change in all random parameters affected the ICER using 10,000 simulations [36]. Beta distributions were used for sampling within the 95% confidence interval of probability variables, gamma distributions for cost variables and lognormal distribution for the remaining parameters. We present these simulation results as cost-effectiveness planes and cost-effectiveness acceptability curves.

## Results

At baseline, the model predicts that across Kenya there will be 32,299 healthcare worker cases and 446 deaths if PPE supply is limited. However, with adequate PPE, only 2189 healthcare worker cases and 30 deaths would be recorded. An extra investment of USD 1.56 million will be required to achieve the reduced number of healthcare worker cases and deaths under the adequate PPE scenario. With this investment, an average of 30,041 healthcare worker cases and 416 healthcare worker deaths will be averted. Overall, a ROI from productivity gains is estimated to be USD 170.64 million, translating into a 11.04 times ROI. Table 2 summarizes the findings from this analysis.

**Table 2:** Summary of cost-effectiveness results^a^

**Incremental Change**			**Cost-effectiveness Ratios**		
HCW Cases Averted	HCW Deaths Averted	Investment (in millions)	Cost per case Averted	Cost per death Averted	Economic Gains (in millions)
30,041 (28,638 - 31,265)	416 (412–418)	$1.55 ($1.54 - $1.55)	$51 ($49 - $54)	$3716 ($3682 - $3748)	$170.64 ($169.34 - $172.09)

95% confidence intervals are derived using estimation of percentiles through a binomial distribution method

*HCW* Healthcare worker

^a^All monetary values are in 2020 US dollars

**Fig. 2:**
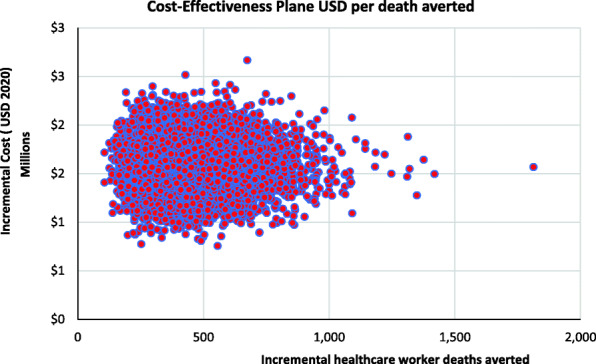
Cost-effectiveness plane for incremental cost per healthcare worker death averted

**Fig. 3:**
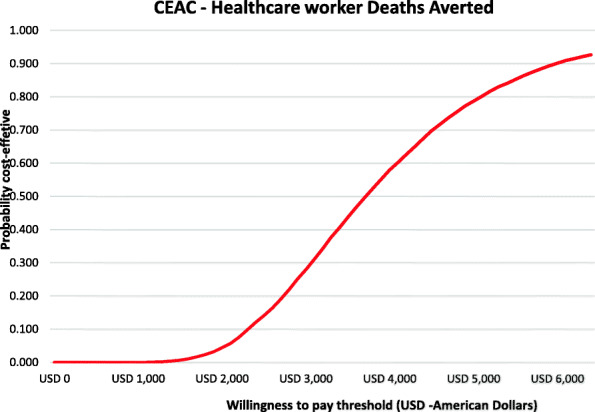
Cost-effectiveness acceptability curve showing the probability that providing adequate PPE is cost-effective in averting a healthcare worker death compared to when healthcare workers are provided with inadequate PPE

Figure 2 shows the cost-effectiveness plane scatter plot for the number of healthcare worker deaths averted in Kenya. All simulated observations indicate that a higher number of healthcare worker deaths are averted when healthcare workers are provided with adequate PPE compared to when the availability of PPE for healthcare workers is limited. However, this would require an additional investment. Figure 3 shows the cost-effectiveness acceptability curve indicating the probability that the scenario with adequate PPE would be cost-effective at averting a healthcare worker death compared to the current scenario where healthcare workers have inadequate PPE over a range of investment values (willingness to pay thresholds). There is a 50, and 75% chance that relative to providing inadequate PPE, investing in adequate PPE would be value for money (cost-effective) if the government or donor would be willing to invest USD 3700, and USD 4800 per averted COVID-19 HCW death.

**Fig. 4:**
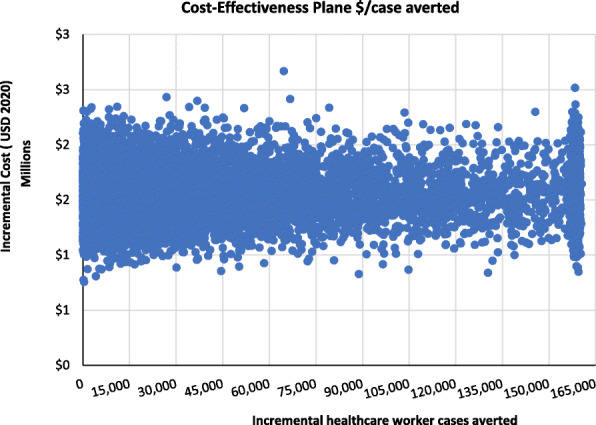
Cost-effectiveness plane for incremental cost per healthcare worker COVID-19 case averted

**Fig. 5:**
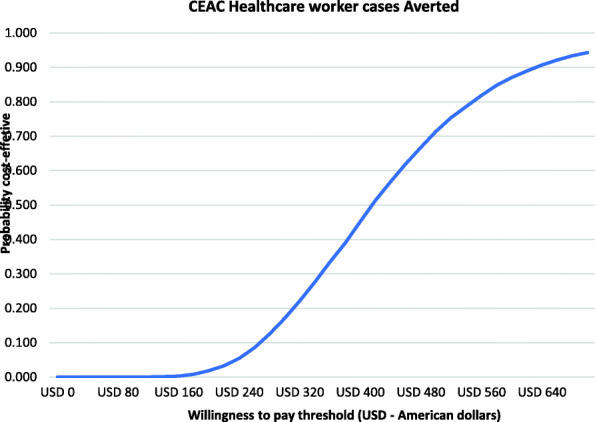
Cost-effectiveness acceptability curve showing the probability that providing adequate PPE is cost-effective in averting a healthcare worker COVID-19 case compared to when healthcare workers are provided with inadequate PPE

Figure 4 shows the cost-effectiveness plane scatter plot for the number of healthcare worker COVID-19 cases averted in Kenya whereas Fig. 5 shows the cost-effectiveness acceptability curve indicating the probability that the scenario with adequate PPE would be cost-effective at averting a healthcare worker COVID-19 case relative to when healthcare workers have inadequate PPE. Relative to inadequate PPE for healthcare workers, investing in adequate PPE would be 25, 50, and 75% cost-effective if the government or donor is willing to pay USD 210, USD 417 and USD 517 per healthcare worker COVID-19 case averted.

## Discussion

This study examined the cost-effectiveness and return on investment (ROI) of protecting healthcare workers in Kenya with personal protective equipment (PPE) during the COVID-19 pandemic using data from Kenya models alongside the WHO ESFT estimates. We found that providing adequate PPE results in 30,041 cases and 416 deaths averted, however, an investment of USD 1.55 million would be required. Such an investment results in a healthcare system gains of USD 170.64 million, equivalent to an 11.04 times ROI.

The higher number of healthcare worker cases and deaths averted is consistent with evidence suggesting that adequate PPE confer some protection against COVID-19 among healthcare workers [2, 5, 37, 38]. Additionally, our findings are similar to those reported by Risko et al. [20].

With the increasing number of COVID-19 cases and healthcare workers contributing over 7% of all cases, there is a need for further investment into PPE. However, this investment should be tied to the enforcement of strict PPE guidelines in the country. Evidence from studies in China showed a decline in the number of cases acquired in the healthcare setting from 41 to 3.8% after enforcing compliance to PPE use [39, 40].

Findings from this study should be interpreted with consideration to the following limitations. First, we omitted several cadres of healthcare workers from the analysis, including community health workers (CHW). Protecting CHW is critical to supporting home-based isolation and care, and other aspects of the pandemic response [41]. Including CHW could have resulted in an even better ROI. Second, not all healthcare workers utilize all PPE daily and our findings may overestimate the investment required to adequately protect all healthcare workers. However, we considered this overestimate as the use of all PPE would reduce the risk of infection among healthcare workers during care encounters. Furthermore, existing evidence supports a zero to low healthcare worker infection rates in countries with stringent PPE compliance [42]. Third, there is a chance that the COVID-19 related mortality in Kenya may be higher than predicted in the model we used as a result of a likely underreporting of COVID-19 deaths in Kenya. If indeed the mortality rate is higher than predicted, then more healthcare worker deaths could be averted for the same investment.

## Conclusion

This analysis provides evidence to inform policy in Kenya and other LMIC of the value of investing in PPE for healthcare workers. Specifically, investing in adequate PPE for protecting all healthcare workers in Kenya has over 10-fold return and would prevent over 70% infection among HCW. We recommend urgent investment into PPEs for health workers but also adherence to the appropriate use of the PPEs.

## Data Availability

The data obtained and analysed in this current study is publicly available [12, 22, 24, 28, 30–32]. The model developed for this analysis can be obtained upon request to the authors (kjacob@kemri-wellcome.org).

## Abbreviations

CHEERS: Consolidated Health Economic Evaluation Reporting Standards
CHW: Community Health Worker
ESFT: Essential Supplies Forecasting Tool
GDP: Gross Domestic Product
HCW: Health Care Workers
ICER: Incremental Cost Effectiveness Ratio
LMICs: Low- and Middle-Income Countries
PPE: Personal Protective Equipment
PSA: Probabilistic Sensitivity Analysis
ROI: Return on Investment
USD: United States Dollars
WHO: World Health Organization

## References

[1] WHO (2020). Over 10 000 health workers in Africa infected with COVID-19.

[2] Nguyen LH, Drew DA, Graham MS, Joshi AD, Guo C-G, Ma W (2020). Risk of COVID-19 among front-line health-care workers and the general community: a prospective cohort study. Lancet Public Health.

[3] Mhango M, Dzobo M, Chitungo I, Dzinamarira T (2020). COVID-19 risk factors among health workers: a rapid review. Saf Health Work.

[4] Organization WH. Risk assessment and management of exposure of health care workers in the context of COVID-19: interim guidance, 19 March 2020. Geneva: World Health Organization; 2020.

[5] Verbeek JH, Rajamaki B, Ijaz S, Sauni R, Toomey E, Blackwood B, et al. Personal protective equipment for preventing highly infectious diseases due to exposure to contaminated body fluids in healthcare staff. Cochrane Database Syst Rev. 2020. 10.1002/14651858.CD011621.pub4. 10.1002/14651858.CD011621.pub4 PMC7158881 32293717

[6] Organization WH (2020). Clinical management of severe acute respiratory infection (SARI) when COVID-19 disease is suspected.

[7] Africa CDC (2020). COVID-19 guidance on use of personal protective equipment for different clinical settings and activities.

[8] World Health Organization. Rational use of personal protective equipment for coronavirus disease (COVID-19) and considerations during severe shortages: interim guidance. Geneva: World Health Organization; 2020.

[9] Burki T (2020). Global shortage of personal protective equipment. Lancet Infect Dis.

[10] MAMO LT (2020). Insights from Africa’s Covid-19 response: repurposing manufacturing.

[11] Mills A (2014). Health care systems in low-and middle-income countries. N Engl J Med.

[12] KNBS (2020). Economic Survey 2020.

[13] KNBS (2020). Statistical Abstract 2020.

[14] Ministry of Health (2015). Kenya Health Workforce Report: The Status of Healthcare Professionals in Kenya, 2015.

[15] Kimathi L (2017). Challenges of the devolved health sector in Kenya: teething problems or systemic contradictions?. Afr Dev.

[16] KENYA MASTER HEALTH FACILITY LIST. 2021 [cited 20/02/2021]. Available from: http://kmhfl.health.go.ke/#/facility_filter/results. Accessed 19 Feb 2021.

[17] Waithaka D, Kagwanja N, Nzinga J, Tsofa B, Leli H, Mataza C (2020). Prolonged health worker strikes in Kenya-perspectives and experiences of frontline health managers and local communities in Kilifi County. Int J Equity Health.

[18] Mbewa DO. More than 3,000 healthcare workers in Kenya test positive for COVID-19. CGTN. 2021. https://africa.cgtn.com/2021/01/11/more-than-3000-healthcare-workers-in-kenya-test-positive-for-covid-19/.

[19] Shilitsa J, Mbenywe M, Kajilwa G. Crisis deepens as doctors join striking medics. London: Standard; 2020.

[20] Risko N, Werner K, Offorjebe A, Vecino-Ortiz A, Wallis L, Razzak J (2020). cost-effectiveness and return on investment of protecting health workers in low-and middle-income countries during the Covid-19 pandemic.

[21] Husereau D, Drummond M, Petrou S, Carswell C, Moher D, Greenberg D (2013). Consolidated health economic evaluation reporting standards (CHEERS) statement. Cost Effect Res Alloc.

[22] Organization WH. COVID-19 essential supplies forecasting tool: frequently asked questions (FAQ), 25 August 2020. Geneva: World Health Organization; 2020.

[23] Organization WH. Rational use of personal protective equipment (PPE) for coronavirus disease (COVID-19): interim guidance, 19 March 2020. Geneva: World Health Organization; 2020.

[24] Barasa E, Kairu A, Nganga W, Maritim M, Were V, Akech S, et al. Examining Unit Costs for COVID-19 Case Management in Kenya. New York: medRxiv; 2020. 10.1136/bmjgh-2020-004159 PMC8053308 33853843

[25] Kenyatta University (2021). Fees Structure.

[26] Study in Kenya (2021). Diploma in Medical Laboratory Sciences at Kenya Medical Training College.

[27] Wakaba M, Mbindyo P, Ochieng J, Kiriinya R, Todd J, Waudo A (2014). The public sector nursing workforce in Kenya: a county-level analysis. Hum Resour Health.

[28] IHME. COVID-19 estimate downloads. 2021. http://www.healthdata.org/covid/data-downloads.

[29] Shange N. Coronavirus infection rate among health workers in SA is 5% — below global average. South Africa: Times; 2020.

[30] Walker P, Whittaker C, Watson O, Baguelin M, Ainslie K, Bhatia S (2020). Report 12: the global impact of COVID-19 and strategies for mitigation and suppression.

[31] Verity R, Okell LC, Dorigatti I, Winskill P, Whittaker C, Imai N (2020). Estimates of the severity of coronavirus disease 2019: a model-based analysis. Lancet Infect Dis.

[32] Guan WJ, Ni ZY, Hu Y, Liang WH, Ou CQ, He JX (2020). Clinical characteristics of coronavirus disease 2019 in China. N Engl J Med.

[33] Kirigia JM, Gbary AR, Muthuri LK, Nyoni J, Seddoh A (2006). The cost of health professionals' brain drain in Kenya. BMC Health Serv Res.

[34] Kenyaadmission (2021). KMTC Fees Structure.

[35] COVID W (2020). Essential supplies forecasting tool.

[36] Briggs A, Sculpher M, Claxton K (2006). Decision modelling for health economic evaluation.

[37] Liu M, Cheng S-Z, Xu K-W, Yang Y, Zhu Q-T, Zhang H (2020). Use of personal protective equipment against coronavirus disease 2019 by healthcare professionals in Wuhan, China: cross sectional study. BMJ.

[38] Zhao Y, Liang W, Luo Y, Chen Y, Liang P, Zhong R (2020). Personal protective equipment protecting healthcare workers in the Chinese epicentre of COVID-19. Clin Microbiol Infect.

[39] Wang D, Hu B, Hu C, Zhu F, Liu X, Zhang J (2020). Clinical characteristics of 138 hospitalized patients with 2019 novel coronavirus–infected pneumonia in Wuhan, China. JAMA.

[40] Zhu S, Zong Z (2020). Why did so few healthcare workers in China get COVID-19 infection. QJM.

[41] Ballard M, Bancroft E, Nesbit J, Johnson A, Holeman I, Foth J (2020). Prioritising the role of community health workers in the COVID-19 response. BMJ Glob Health.

[42] Neuwirth MM, Mattner F, Otchwemah R (2020). Adherence to personal protective equipment use among healthcare workers caring for confirmed COVID-19 and alleged non-COVID-19 patients. Antimicrob Resist Infect Contr.

